# Subclinical Presentation of Cutibacterium acnes Infection Following Revision Rotator Cuff Surgery

**DOI:** 10.7759/cureus.80080

**Published:** 2025-03-05

**Authors:** Zachary Walker, David Shuster, David Sosnoski, Ali Hamade, Marc Milia, Hussein Saad

**Affiliations:** 1 Orthopedic Surgery, Corewell Health Farmington Hills, Farmington Hills, USA; 2 Orthopedic Surgery, Corewell Health Dearborn, Dearborn, USA

**Keywords:** arthroscopic rotator cuff repair, cutibacterium acnes, orthopedic arthroscopy, rotator cuff repair surgery, shoulder septic arthritis

## Abstract

We present a unique case of an individual who presented with a culture-proven *Cutibacterium acnes* (C. acnes) infection 2.5 months after a revision arthroscopic rotator cuff repair. Instead of waiting for the result of aspiration cultures and consequently delaying surgery, we decided to take the patient to the operating room for arthroscopic irrigation and debridement on the date of presentation. Our purpose is to add to the existing evidence of C. acnes septic arthritis and, more specifically, to highlight the subclinical nature of this infection and our prompt decision to take the patient to the operating room for irrigation and debridement without culture-proven infection.

## Introduction

*Cutibacterium acnes *(C. acnes) is a gram-positive anaerobic bacillus, which is commonly found in the sebaceous glands of the skin, generally in the axilla, chest and back regions [1,2]. This results in an increased prevalence of C. acnes around the shoulder versus other orthopedic surgical sites. Although C. acnes is normal skin flora, surgical incisions bring on the possibility of inoculation to the shoulder joint causing infectious complication. Shoulder infections have been reported to occur at 3.4% following arthroscopic or open shoulder surgeries, where they are most commonly due to C. acnes [3,4]. Although this number is relatively low, it is still significant enough to cause morbidity and negate improvement in a great deal of patients, namely patients following rotator cuff repair [5,6]. Postsurgical infections that are characterized by erythema, purulence, cellulitis and severe pain are more easily diagnosed. However, low-grade, low-virulence infections are much more difficult to diagnose and should be considered in patients with a more subtle clinical course. The diagnostic difficulty is due to the vague, delayed presentation, mildly elevated and/or normal laboratory inflammatory markers, and the inability to correctly draw and interpret joint fluid cultures [7,8].

Although literature has been reported on the emergence of C. acnes as a considerable postoperative etiology, the exact subclinical presentation following a revision rotator cuff repair has been limited [1,7]. We present an otherwise healthy male patient who was successfully treated for a subclinical C. acnes infection following revision rotator cuff repair with arthroscopic irrigation and debridement along with antibiotics.

## Case presentation

Our case involves a 54-year-old right-hand dominant male who presented with left shoulder pain. We first met this patient when he presented to the orthopedic clinic 14 years after undergoing left shoulder arthroscopy with rotator cuff repair. He was otherwise a relatively healthy, active male, with a past medical history significant for hypertension, sleep apnea, and polycythemia vera. 

**Figure 1:**
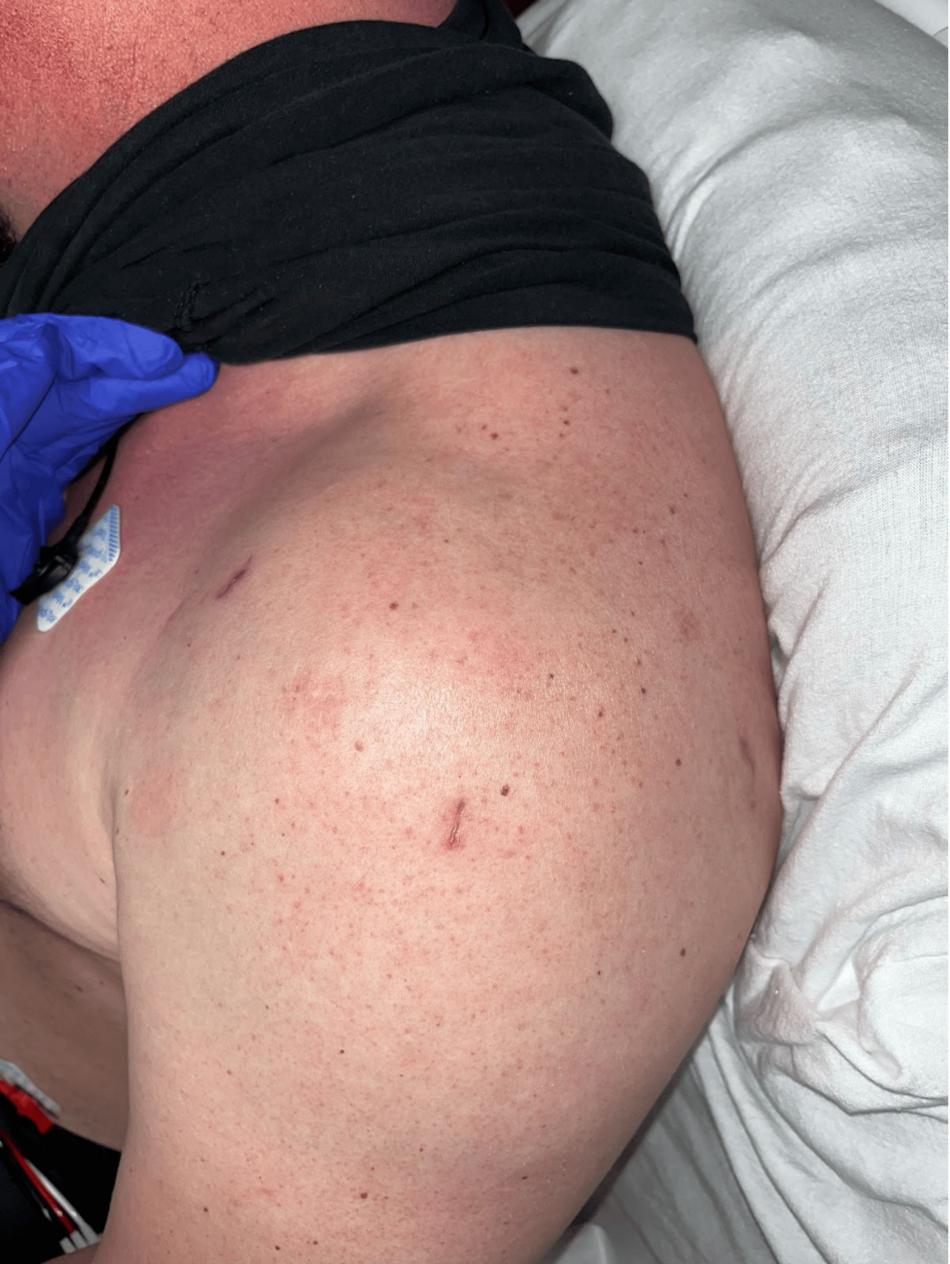
Presentation of the affected shoulder Notice the lack of erythema and well-healed incisions. Clinically it does not appear like a septic shoulder.

At his initial presentation to our orthopedic clinic, on exam, he exhibited positive Jobe test, drop arm sign, along with decreased range of motion overhead secondary to pain. MRI demonstrated a full thickness supraspinatus and infraspinatus tear, which he likely sustained while working at his labor-intensive job, though there was no specific inciting injury. He subsequently underwent left shoulder arthroscopy with revision rotator cuff repair utilizing suture tape and two push-lock suture anchors. The shoulder was prepared with hydrogen peroxide followed by chlorhexidine, and draped in the usual sterile manner in the beach chair position. He started normal rotator cuff repair postoperative protocol and progressed well for the initial two weeks. The patient had a slow progression during his postoperative rehabilitation course, but otherwise was uneventful as he regularly participated in physical therapy. Approximately three months following the procedure, he developed one week of increased left shoulder pain without injury and persistent fever. Temperature was 102°F on arrival. On physical exam, he had full, painless range of motion of the left shoulder. The surgical incisions were healed without erythema, and there wasno tenderness to palpation with no appreciable effusion (Figure 1).

**Figure 2:**
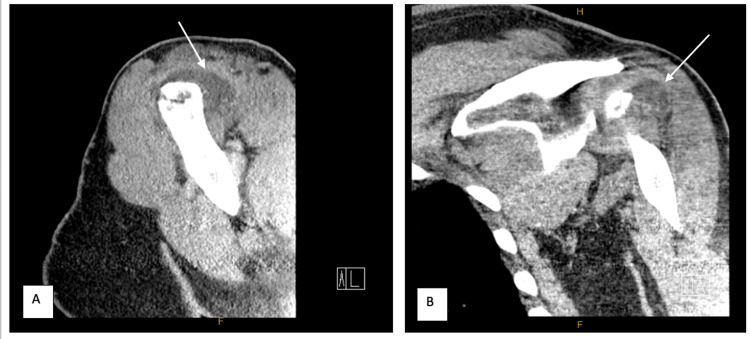
Axial (A) and coronal (B) CT with contrast images of the left shoulder demonstrating a distended subacromial bursa Arrows in A and B point to the distended subacromial bursa.

Upon presentation to the hospital, he demonstrated negative Jobe test, and positive Hawkin’s impingement testing. Otherwise, his shoulder exam was benign. Initial lab workup returned with white blood cell (WBC) count 10.9 bil/L (normal: 4-11 bil/L), C-reactive protein (CRP) 23 mg/L (normal: <10 mg/L), and erythrocyte sedimentation rate (ESR) 13 mm/hr (normal: 0-15 mm/hr). These labs are below or near normal reference ranges. A CT scan with IV contrast of the left shoulder was ordered, which showed a distended subacromial bursa otherwise no abscess, fracture or osteomyelitis (Figure 2). The subacromial space was then prepped with chlorhexidine scrub and aspirated with 18-gauge needle in sterile fashion. This yielded 20 mL of dark-red, bloody fluid. The aspirate was sent to the lab and was negative on initial gram stain and crystal analysis. Interestingly, the total nucleated cell count was 84,800 (normal: <50,000 cells) with 300,000 red blood cells (no normal reference). Given his high nucleated cell count, fevers, shoulder pain and CT results, we had a high clinical suspicion for subclinical left shoulder septic arthritis. As a result, we believed this to be a case of C. acnes septic arthritis, and made the decision to proceed to the operating room for arthroscopic irrigation and debridement without culture proven infection. 

**Figure 3:**
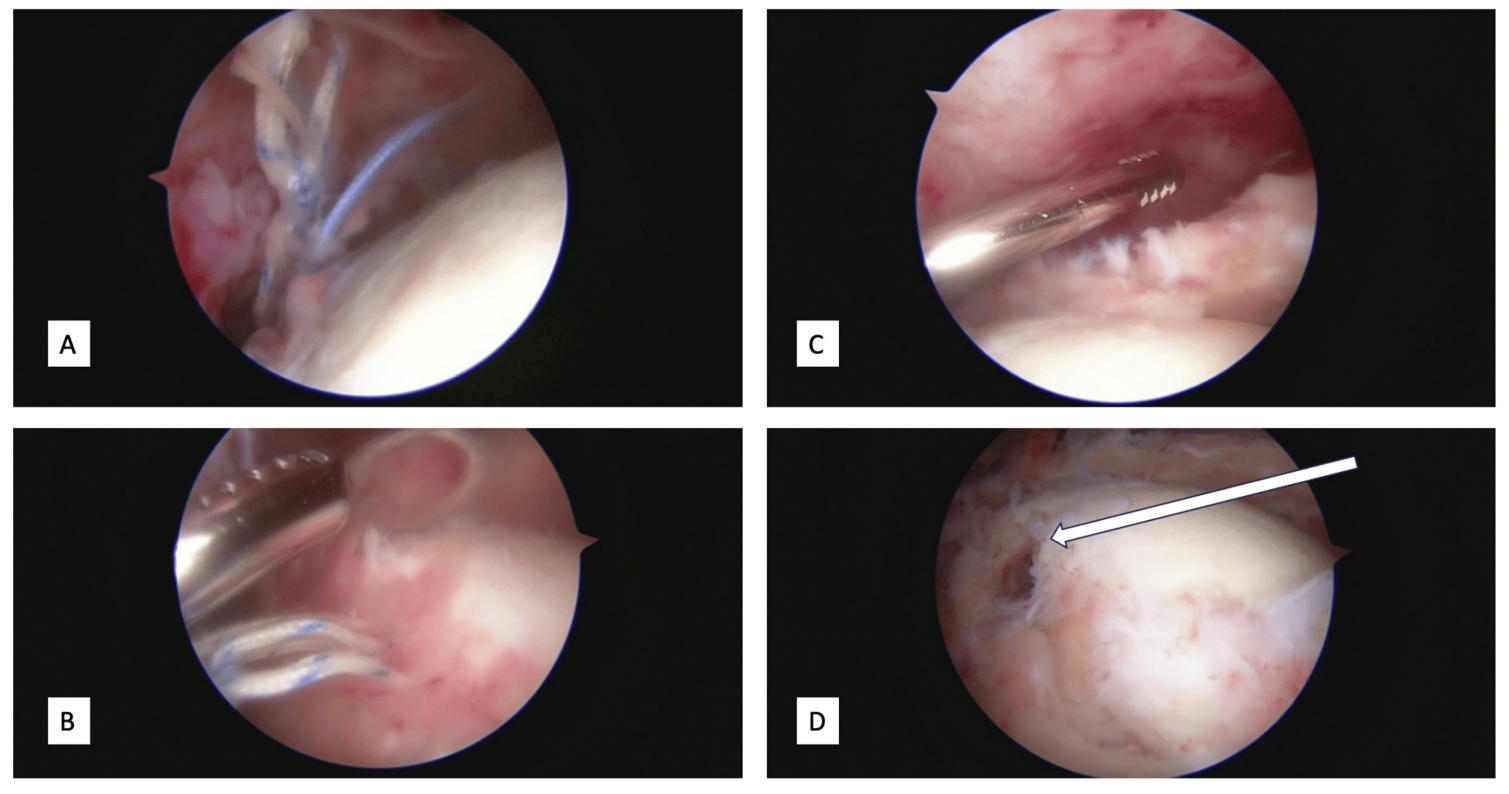
Arthroscopic images of the left shoulder A, B demonstrate the detached sutures floating within the joint consistent with re-tear of the rotator cuff; C demonstrates the retracted, torn rotator cuff beneath the arthroscopic shaver; D shows an arrow pointing to the hole in the humerus demonstrating where the bioabsorbable suture anchor used to be.

Intraoperatively, upon entering the glenohumeral joint, viscous dark serosanguinous fluid was encountered and taken for culture. It is difficult to say whether it was purulent or not given its color, though it clearly did not appear to be normal joint fluid. The supraspinatus and infraspinatus were found to be completely torn with torn sutures still attached to the tendons. The considerable amount of friable tissue was removed with a shaver followed by irrigation. The sutures and previous bioabsorbable anchors were all removed (Figure 3). 

Once the procedure was completed, he was placed in a sling for comfort. Postoperatively, the patient unfortunately developed pneumonia, and was started on dual IV antibiotic therapy with vancomycin and ceftriaxone to cover his shoulder as well. He was discharged on postoperative day 4 with six weeks of vancomycin and ceftriaxone. On day 13 postop, two of two separate intraoperative fluid samples were found positive for C. acnes. The fluid aspiration taken in the emergency room did not grow anything on final cultures. He then continued IV ceftriaxone 2 g daily for six weeks postoperatively, along with formal physical therapy. At the eight-week postoperative visit, he had no pain on physical exam with full range of motion, minimal supraspinatus and infraspinatus weakness, and returned to work and daily activities as tolerated at that time. Upon discussion with the patient, the patient declined any further surgical intervention and desired to pursue continued conservative management with physical therapy.

## Discussion

C. acnes is a substantial bacterial pathogen in patients undergoing open and arthroscopic shoulder surgery, with multiple studies reporting this bacterium to be responsible for over 50% of postoperative infections [5,9-12]. It has been reported that 7% to 29% of shoulder surgery cases were positive for residual C. acnes following skin preparation, and greater than 40% of shoulder cases will have culture-positive skin cultures at the case’s conclusion [13,14]. Suture contamination has also been reported to be a culprit causing indolent C. acnes infection, and a multitude of studies have looked at different skin preparations including chlorhexidine, hydrogen peroxide, and others, all reporting different results [15-19].

With lower virulence, the presentation of patients plagued with a C. acnes infection can be subclinical, displaying vague clinical symptoms, inflammatory markers that have been reported to be typically within normal limits, and inability to correctly draw and interpret synovial fluid cultures [7,8,20]. Malige et al. reported that greater than 25% of patients undergoing revision rotator cuff repair had a subclinical shoulder infection, with 17 of 21 patients positive for C. acnes alone. They also described cultures returned positive on average of 13.52 days following the original cultures being taken [21]. Similarly, another study demonstrated a subclinical infection rate of 27.2% following arthroscopic shoulder stabilization surgery [22]. The result of the culture came back at 13 days after the initial collection in our presentation, which is similar to the reported median time from specimen collection of 10 (4-24) days to positive culture [23].

It is possible that the positive culture for C. acnes postoperative day 13 was a contaminant; however, we do not believe this to be the case. The two cultures that resulted positive were taken from the joint fluid, which was taken in the operating room under sterile conditions. Additionally, the patient's rotator cuff was found to be torn with no inciting trauma. Infection is a known cause for rotator cuff repair failure [21]. 

Our case presentation represents a patient with subclinical C. acnes infection similar to those reported by Malige et al. and Horneff et al. [21,24]. In both studies, they were unable to identify direct risk factors associated with the development of subclinical infections; however, another study identified males having a five times higher likelihood to be culture positive [22]. Yeranosian et al. also identified older age to be a risk factor for developing infection needing reoperation; however, they concluded the age group greater than 60 at most risk [12]. In contrast, we presented a 54-year-old male in our case. Yeranosian et al. also reported on patients requiring reoperation within 30 days, and our report includes a patient requiring an operation approximately 90 days after surgery [12]. The subclinical presentation with an infectious etiology cannot be overlooked even three months postoperatively. Proper workup is essential to mitigate a patient who may present late in their postoperative follow up with a subpar course. A high index of suspicion and prompt treatment, much like in the our case presentation, can successfully treat and eradicate a C. acnes-positive subclinical infection to improve the clinical outcome in a difficult patient population.

## Conclusions

We present an interesting report of C. acnes-positive joint infection three months following a revision arthroscopic rotator cuff repair in a relatively healthy patient. We decided to take the patient to the operating room prior to culture proven infection and this resulted in an expedited and complete recovery in our patient. Our case presentation reiterates the necessity for an appropriate work up in patients with a suboptimal clinical course. This patient represents a difficult patient population to treat; however, with a high index of clinical suspicion, these treatable indolent infectious complications following revision rotator cuff surgery can be properly managed with prompt irrigation, debridement, and antibiotic regimen.
